# Doctors' Wills

**Published:** 1914-05-16

**Authors:** 


### Doctors' Wills.

Dr. John Ellicott, M.D., of Greentown, Florencecourt, County Fermanagh, has left personal estate valued at £1,926.

Sir Francis McCabe, F.R.C.P., M.R.C.S., of Sandyford, County Dublin, who was formerly Medical Commissioner of the Local Government Board for Ireland, has left estate valued at £8,725.

Mr. George Ernest Herman, M.B., F.R.C.P., F.R.C.S., of Carn, Glos., late of 20 Harley Street, W., formerly consulting obstetric physician to the London Hospital, etc., examiner in midwifery to the Royal College of Surgeons and Physicians and to the Universities of Oxford, Cambridge, London, and Durham, left estate of which £7,149 is net personalty.

